# Supplementary material for: Allosteric control of dynamin-related protein 1 through a disordered C-terminal Short Linear Motif
Source: Nat Commun. 2024 Jan 2;15:52. doi: 10.1038/s41467-023-44413-6 (PMC10761769; doi:10.1038/s41467-023-44413-6)
Supplement: Supplementary file 1 — Supplementary Information [file 41467_2023_44413_MOESM1_ESM.pdf]

Allosteric control of dynamin-related protein 1-catalyzed mitochondrial and peroxisomal fission through a conserved disordered C-terminal Short Linear Motif

Pérez-Jover et al.

Supplementary Information

Supplementary Figures

a

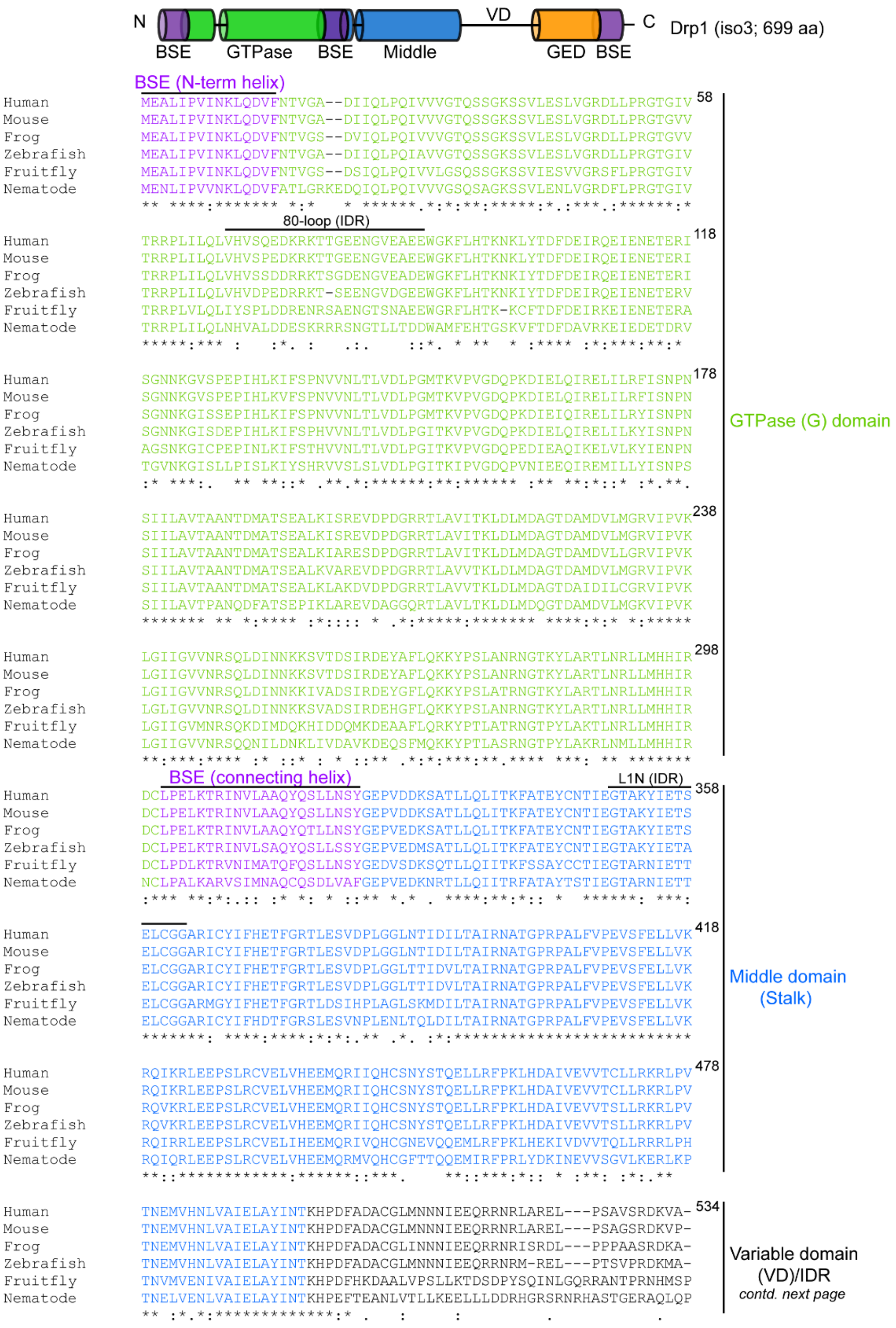

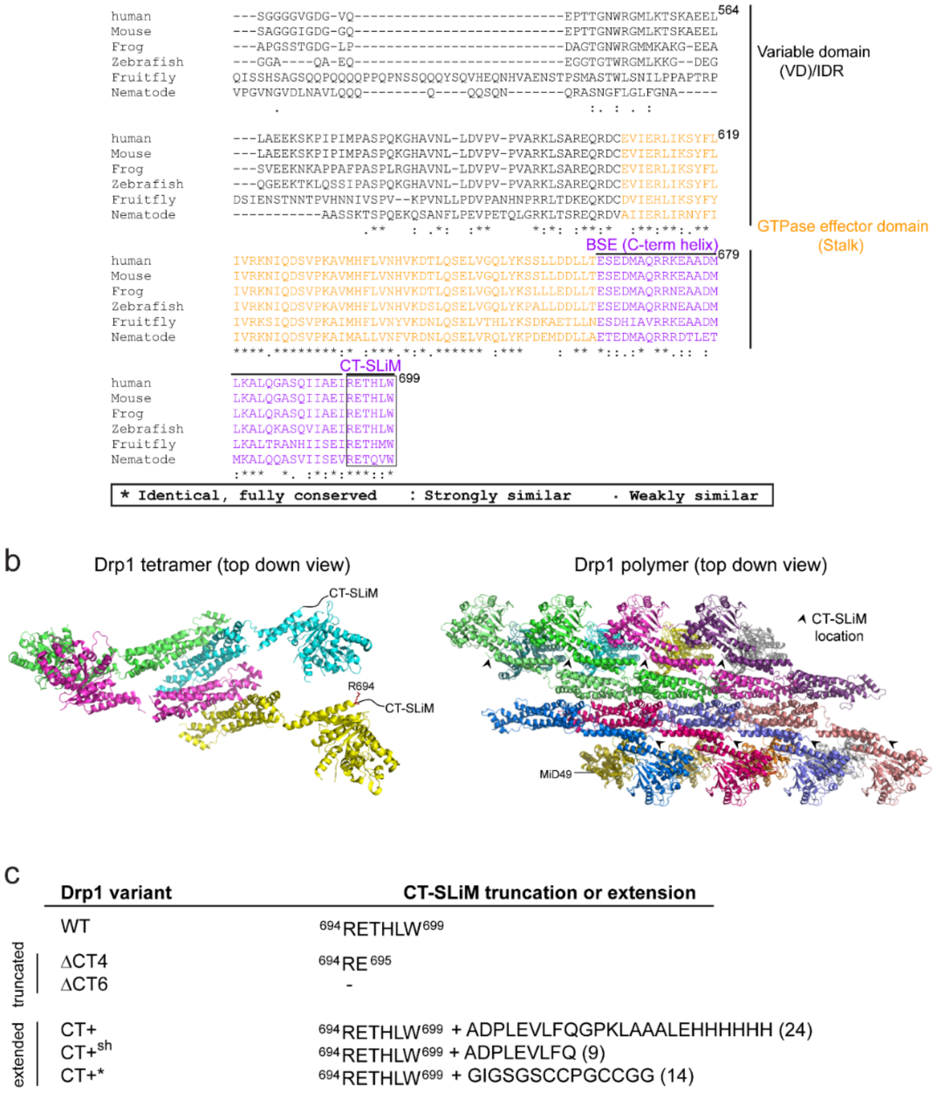

Supplementary Figure 1. CT-SLiM conservation in metazoans, its location at a critical inter-subunit interface in the helical oligomer, and the various CT-SLiM modifications used in the study. a Clustal Omega (1.2.4) multiple sequence alignment of human Drp1 (from the ubiquitously expressed and shortest 699 aa isoform 3 used in the study; NCBI Reference Sequence: NP_005681.2) with that of mouse (*Mus musculus*; NP_001021118.1), frog (*Xenopus laevis*; NP_001080183.1), zebrafish (*Danio rerio*; AAH55521.1), Fruitfly (*Drosophila melanogaster*; NP_608694.2, and nematode (*Caenorhabditis elegans*; AAD49861.1). Sequences are color-coded in correspondence with the cartoon illustration of the Drp1 primary structure above. The location of the various prominent IDRs is indicated above. **b** Top-down views of the Drp1ΔVD tetramer (PDB ID: 4BEJ; left) and of the full-length Drp1 oligomer in association with the soluble domain of adaptor MiD49 (PDB ID: 5WP9; right) showing the location of the CT-SLiM. In the oligomer structure, the resolved CT portion is located adjacent to the BSE-stalk interface of the neighboring subunit as indicated by arrows. **c** Polypeptide sequences of the various CT modifications used in this study. See Methods for additional details.

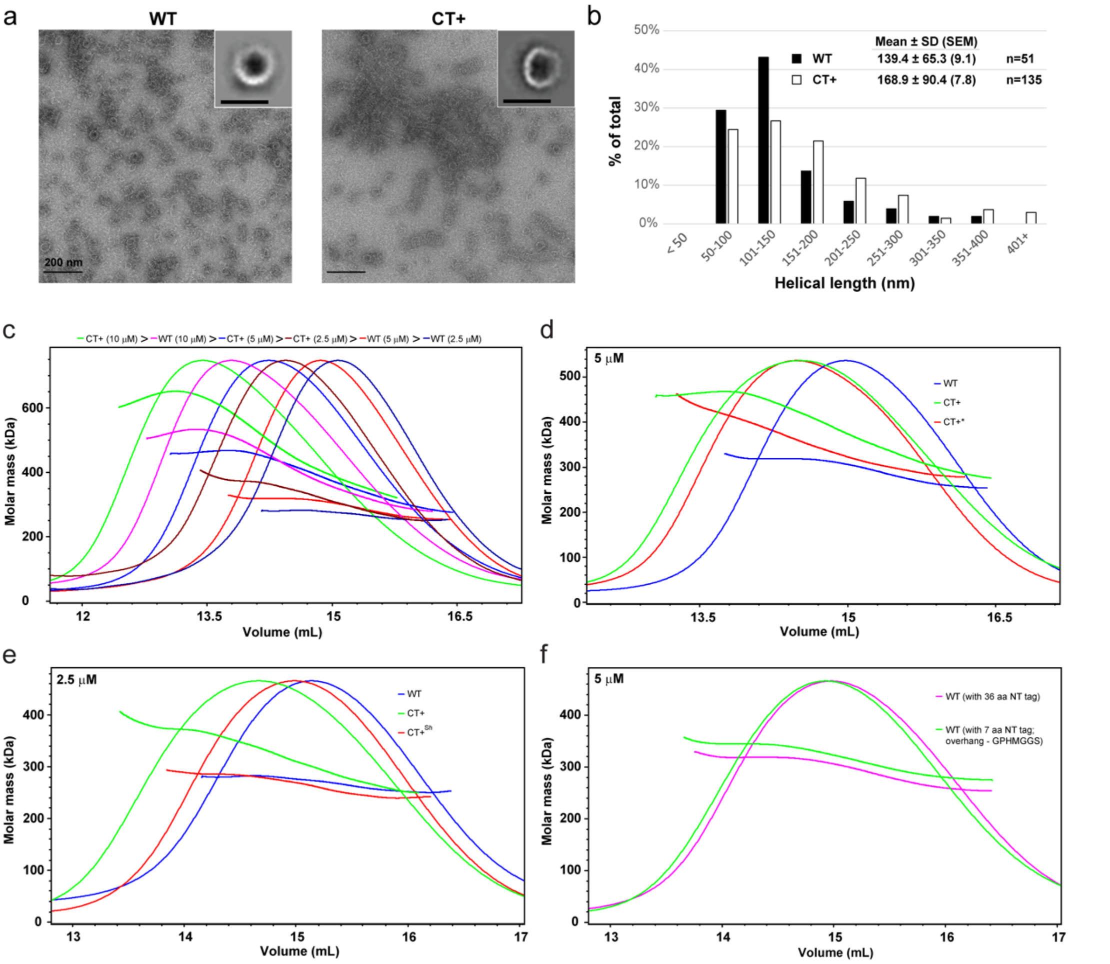

Supplementary Figure 2. NS-EM and SEC-MALS analysis of CT variants. a Representative NS-EM images of human WT Drp1 and the CT+ Drp1 variant in the presence of the non-hydrolyzable GTP analogue, GMP-PCP. Scale bar, 200 nm. Insets show 2D class averages of the predominant oligomer (ring) morphology. Insets scale bar, 50 nm. **b** Histograms showing the distribution of assessed helical polymer length for WT Drp1 versus human CT+ Drp1. **c-f** Comparison of SEC-MALS profiles of human WT Drp1 containing a 36 aa residue N-terminal His_6 tag with: **c** CT+ Drp1 (mouse) at 10 μM, 5 μM, and 2.5 μM injection concentrations, **d** with human WT Drp1 containing a much shorter 7 aa residue tag at the N-terminus, **e** with CT+ Drp1 (mouse) and a N-terminally His_6 tagged CT+* Drp1 (human), and **f** with CT+ Drp1 (mouse) and CT+^{sh} Drp1 containing a much shorter 9 aa residue CT extension. See Supplementary Figure 1c and Methods for additional details. For panels **d-f**, the protein concentration at injection is indicated.

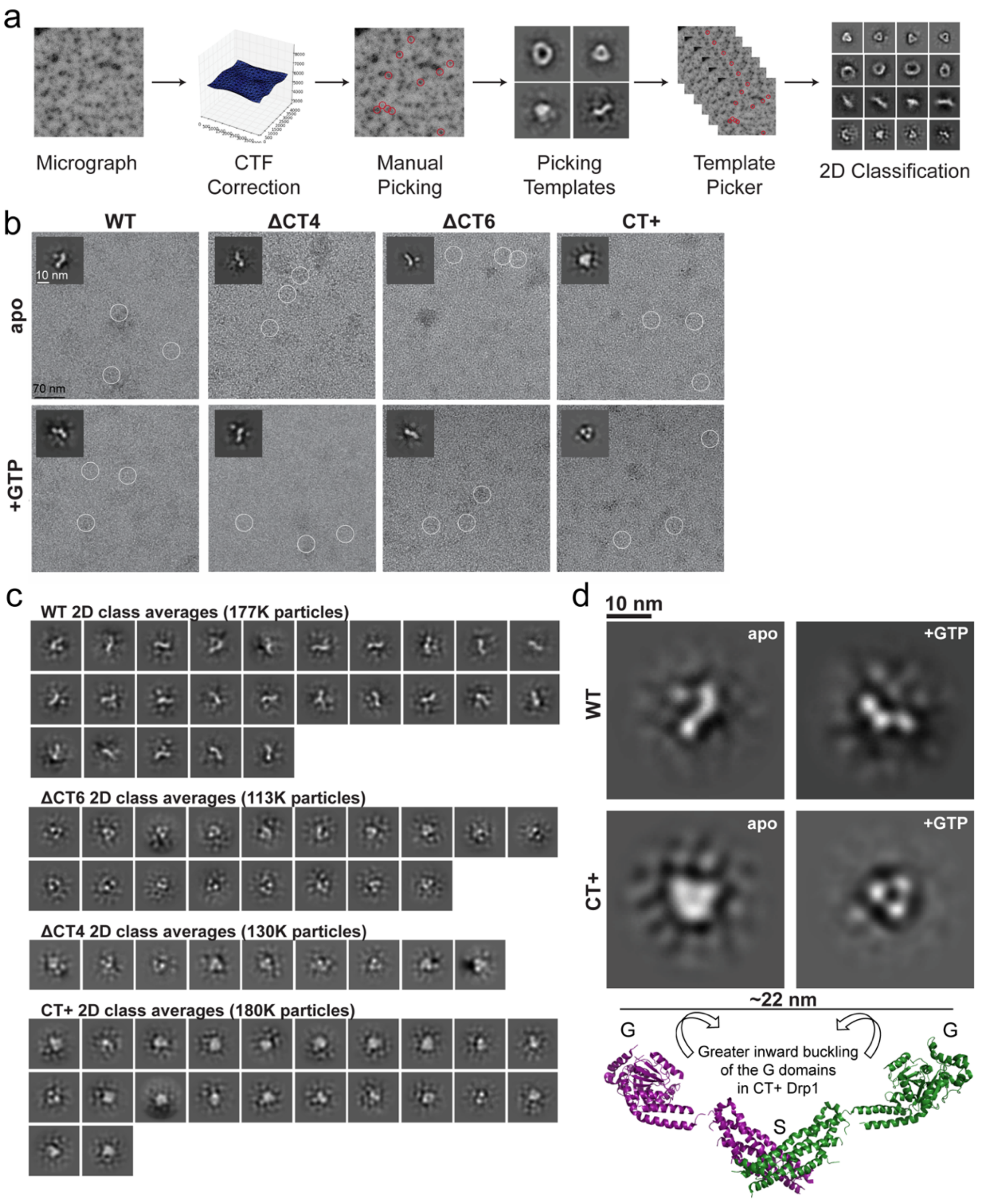

Supplementary Figure 3. NS-EM 2D classification workflow and analysis of CT variants under different states. **a** Workflow of NS-EM 2D classification. See Methods for further details. **b** Analysis of dimer conformation for human WT Drp1 and CT variants in the apo and GTP hydrolysis states from NS-EM micrographs. Scale bar, 70 nm. The diameter of the circles is 40 nm. Insets show the predominant 2D class averages. Inset scale bar, 10 nm. **c** 2D class averages for WT and CT variants in the apo state after one round of refinement. The number of particles utilized for each were: WT = 177K, ΔCT4 = 130K**,** ΔCT6 = 113K, and CT+ Drp1 = 180K. **d** Close-up views of WT Drp1 and CT+ Drp1 dimers (2D class averages) in the apo and GTP hydrolytic states in solution. The compact conformation of CT+ Drp1 versus the extended conformation of WT Drp1 likely results from a pronounced inward buckling of the two

G domains toward each other in CT+ Drp1 as shown at the bottom. G is G-domain and S is stalk.

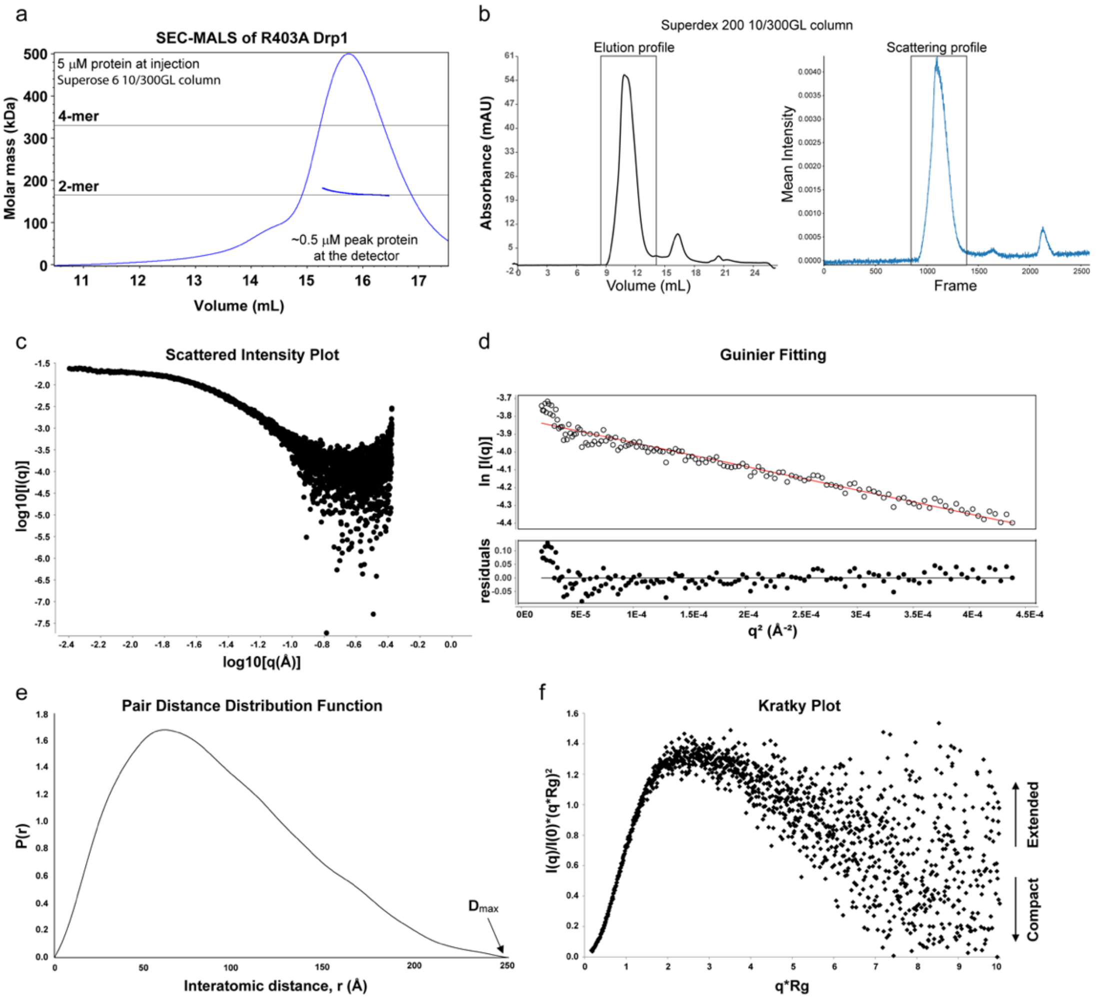

Supplementary Figure 4. SAXS analysis of dimeric R403A Drp1. a SEC-MALS analysis of R403A Drp1. **b** SEC-SAXS profiles of R403A Drp1. **c, d** SAXS curves in reciprocal (Fourier) space (**c**) with a linear Guinier region (**d**) demonstrate sample homogeneity. The radius of gyration (R_g) calculated from the Guinier plot for R403A Drp1 was 60.74 ± 1.52 Å. **e** Pair-distance distribution function (PDDF) was calculated from the scattering data using indirect Fourier transform in GNOM module from ATSAS package. The maximum dimension of the protein, D_{max}, was found to be ~230 Å. **f** Normalized Kratky plot for R403A Drp1 showing bell-shaped profiles as well as plateau at large scattering values indicate a well-folded protein with globular domains alongside highly flexible regions.

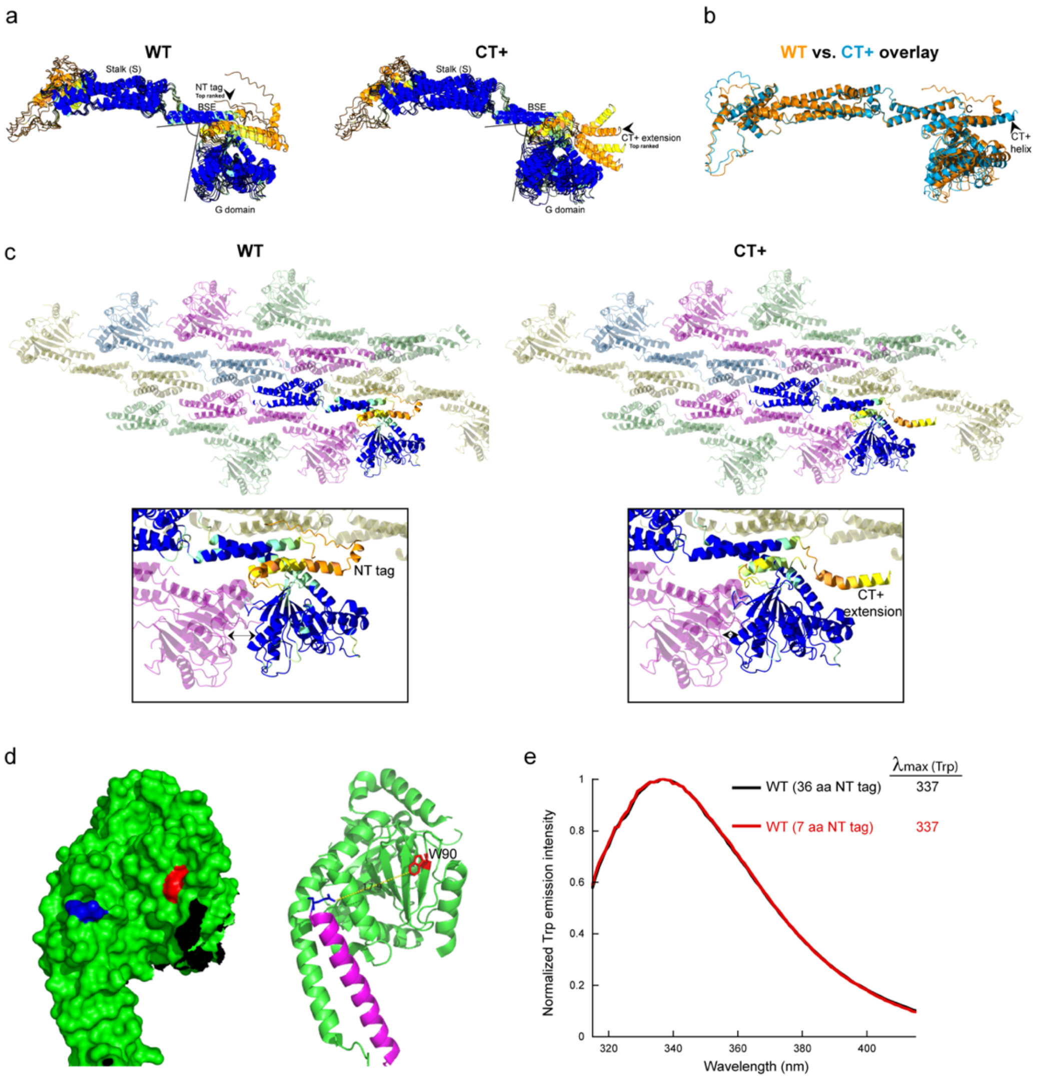

Supplementary Figure 5. AlphaFold modeling and iTFS reveal distinct conformational alterations in CT+ Drp1. a AlphaFold models of N-terminally tagged WT Drp1 and C-terminally tagged CT+ Drp1 following the AFDB colouring scheme, with blue representing high pLDDT and orange representing low pLDDT. The top-ranked conformer is indicated near the corresponding affinity tags or pointed by an arrow. Note that the non-native sequence extension in CT+ Drp1 propagates as an α-helix and juxtaposes with the G domain, causing a noticeable inward buckling of the G domain toward the BSE. The N-terminal tag, on the other hand, is mostly disordered and does not appear to contact the top of the G domain. **b** Overlay of the top-ranked conformers of N-terminally tagged WT and C-terminally tagged CT+ Drp1. The C-terminus at the end of the terminal BSE helix and the non-native CT+ helix that extends beyond are indicated. **c** Superposition of the AlphaFold-derived monomer structures for WT Drp1 and CT+ Drp1 on the Drp1 oligomer cryo-EM structure (PDB ID: 5WP9). One monomer in the cryo-EM structure is replaced by an AFDB scheme-colored AlphaFold structure. Note that the G domain in CT+ Drp1 is more pronouncedly buckled toward the G domain of the neighboring subunit on its left relative to WT Drp1 (denoted by the shrinking of a double-headed arrow placed in between the two G domains). Insets show zoomed-in views of the altered conformation and bucking of the CT+ Drp1 G domain relative to WT Drp1. **d** Surface and ribbon representations of the

G domain and BSE showing the proximity of W90 (red) in the G domain to the last resolved residue (I693, blue) in the BSE C-terminal helix (magenta). Distance between W90 and I693 was calculated to be 17.9 Å, within Trp-Trp homo-FRET distance (~24 Å) to adjacent but unresolved W699. **e** Normalized Trp emission spectra of two human WT Drp1 constructs containing a 36 aa-residue versus a 7 aa-residue N-terminal tag. λ_{max} indicated above shows no effect of tag length and sequence on Trp emission intensity originating from W699 in the CT-SLiM.

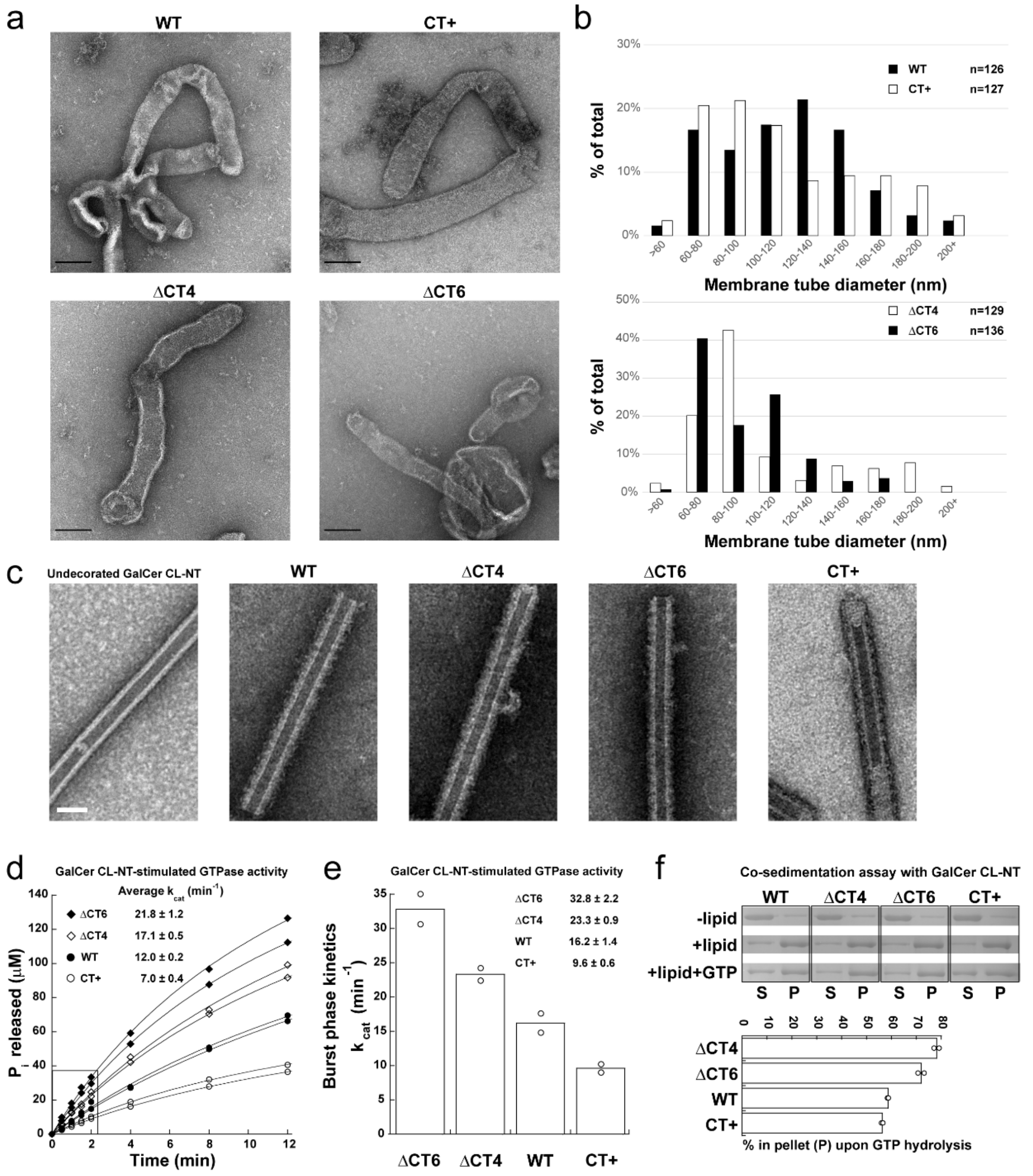

Supplementary Figure 6. All CT variants retain the membrane remodeling and assembly capacities of WT Drp1 but are divergent in activity. a NS-EM of WT Drp1 and CT variants on CL-containing liposomes. Scale bar, 200 nm. **b** Histograms showing the distribution of membrane tube diameters for WT Drp1 and the CT variants. **c** NS-EM of WT Drp1 and CT variants on GalCer CL-NTs. Scale bar, 50 nm. **d** CL-stimulated GTPase activities of WT Drp1 and CT variants at 0.5 μM each in the presence of GalCer CL-NT (150 μM total lipid). The concentration of inorganic phosphate (P_i) released is plotted against time. Individual data points and fitted traces from two independent experiments (n = 2) are shown. Mean ± SEM is indicated above. **e** k_{cat} for the burst-phase pre-steady-state kinetics only from panel (**d**) (boxed area). Individual data points and averages (bars) from the two independent experiments are shown. **f** Co-sedimentation analyses of WT Drp1 and CT variants in the absence and presence of GalCer CL-NTs, and upon GTP addition to GalCer CL-NT-preincubated Drp1 samples (one experiment for each condition). S refers to supernatant and P refers to pellet. % Drp1 in pellet upon GTP hydrolysis on GalCer CL-NTs is presented as a percentage of total with errors in the densitometric analysis of the bands plotted.

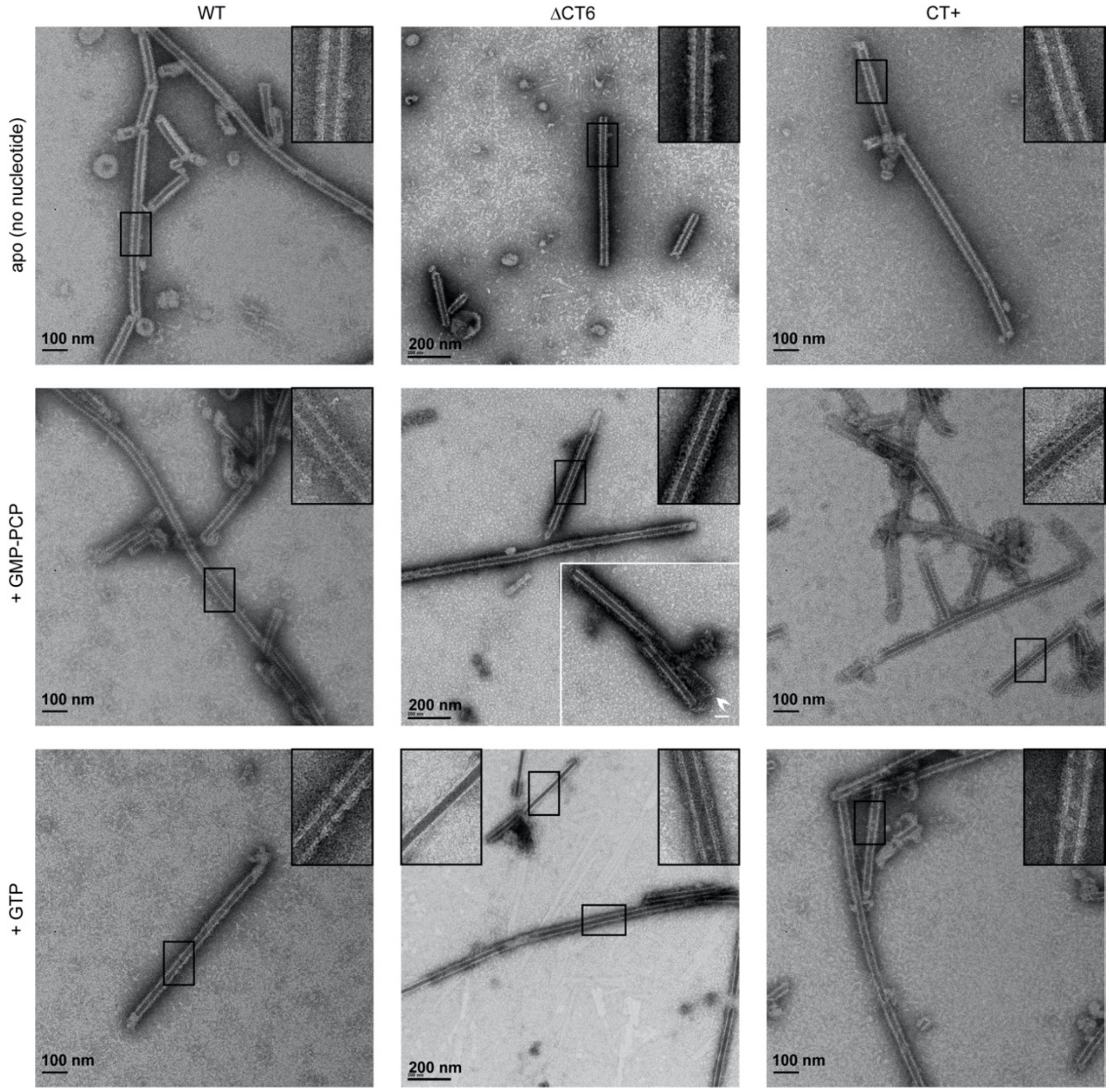

Supplementary Figure 7. WT and CT variants are differentially capable of assembly and disassembly on GalCer CL-NTs. NS-EM of WT Drp1 (left column), ΔCT6 (middle column), and CT+ Drp1 (right column) assembled on GalCer CL-NTs in the apo state (top row), and upon incubation with GMP-PCP (middle row) or GTP (bottom row) post assembly. Scale bar, 100 or 200 nm as indicated. Boxed regions are zoomed and shown as insets. White arrowhead in the ΔCT6 + GMP-PCP panel points to a helical polymer that seemingly extends beyond the lipid template as seen sporadically with this variant under this condition. Insets in the ΔCT6 + GTP panel show both bare NTs from which the variant presumably has completely disassembled, as well as fully decorated NTs on which compactly organized helical polymers of this variant persist.

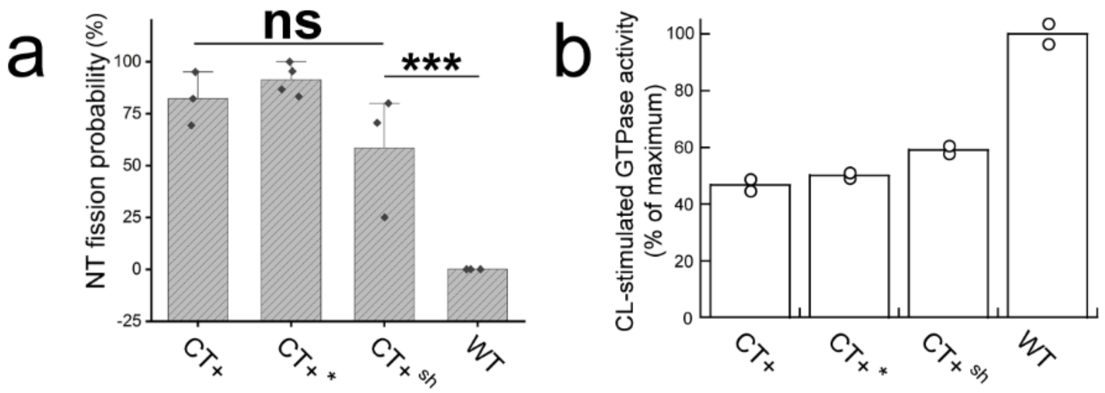

Supplementary Figure 8. Increased NT fission correlates with suppression of GTPase activity. a NT fission activity of the CT+ variants (154 NTs for CT+, 80 NTs for CT+*, 45 NTs for $CT+^{sh}$) compared to WT Drp1 (154 NTs). Each point represents an independent experiment. Mean ± SD are shown. ns - not significantly different ($p > 0.05$), *** significantly different at 0.001 level (unpaired two sample t-test). n = 3, except for CT+*, where n = 4. **b** Relative CL-stimulated GTPase activities of the CT+ variants shown as a percentage of the maximum exhibited by WT Drp1. Individual data and averages (bars) from two independent experiments (n = 2) are shown.

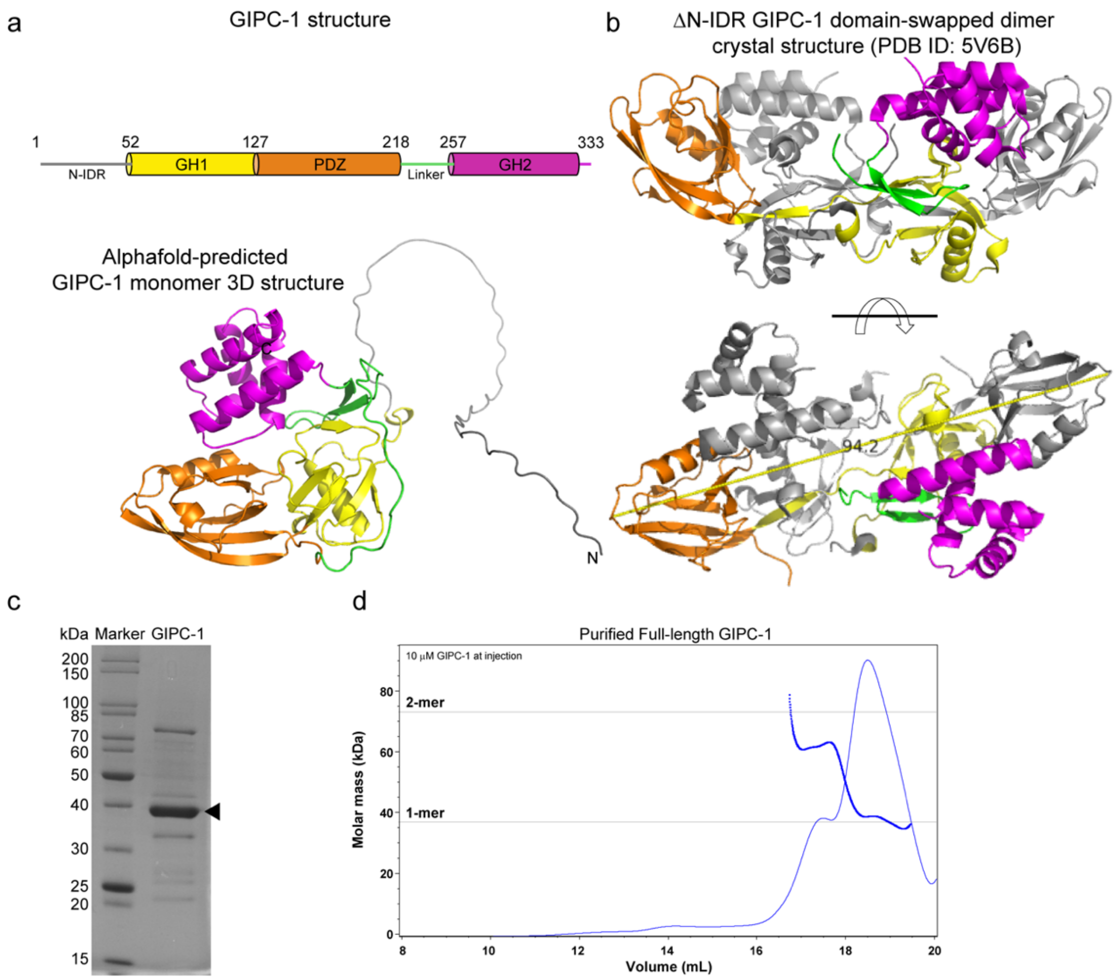

Supplementary Figure 9. Structural and biochemical characterization of full-length GIPC-1. a Cartoon representation of domain arrangement in the GIPC-1 primary structure, and a corresponding color-coded AlphaFold model of the full-length GIPC-1 monomer. **b** Crystal structure of the PDZ domain-swapped GIPC-1$^{\Delta N51}$ dimer (PDB ID: 5V6B). One of the two monomers is color-coded in correspondence to the structures shown in panel A. Top and side views are shown. Distance between the target motif (PBM) binding sites of the two PDZ domains was found to be ~94.2 Å. **c** Representative SDS-PAGE of full-length GIPC-1 after GST excision. **d** SEC-MALS analysis of purified, full-length GIPC-1 injected at 10 μM and sieved using a Superdex 200 10/300 GL column. Molar masses corresponding to monomer (1-mer) and dimer (2-mer) states are indicated by horizontal lines. An intermediate mass reveals a fast monomer-dimer equilibrium.

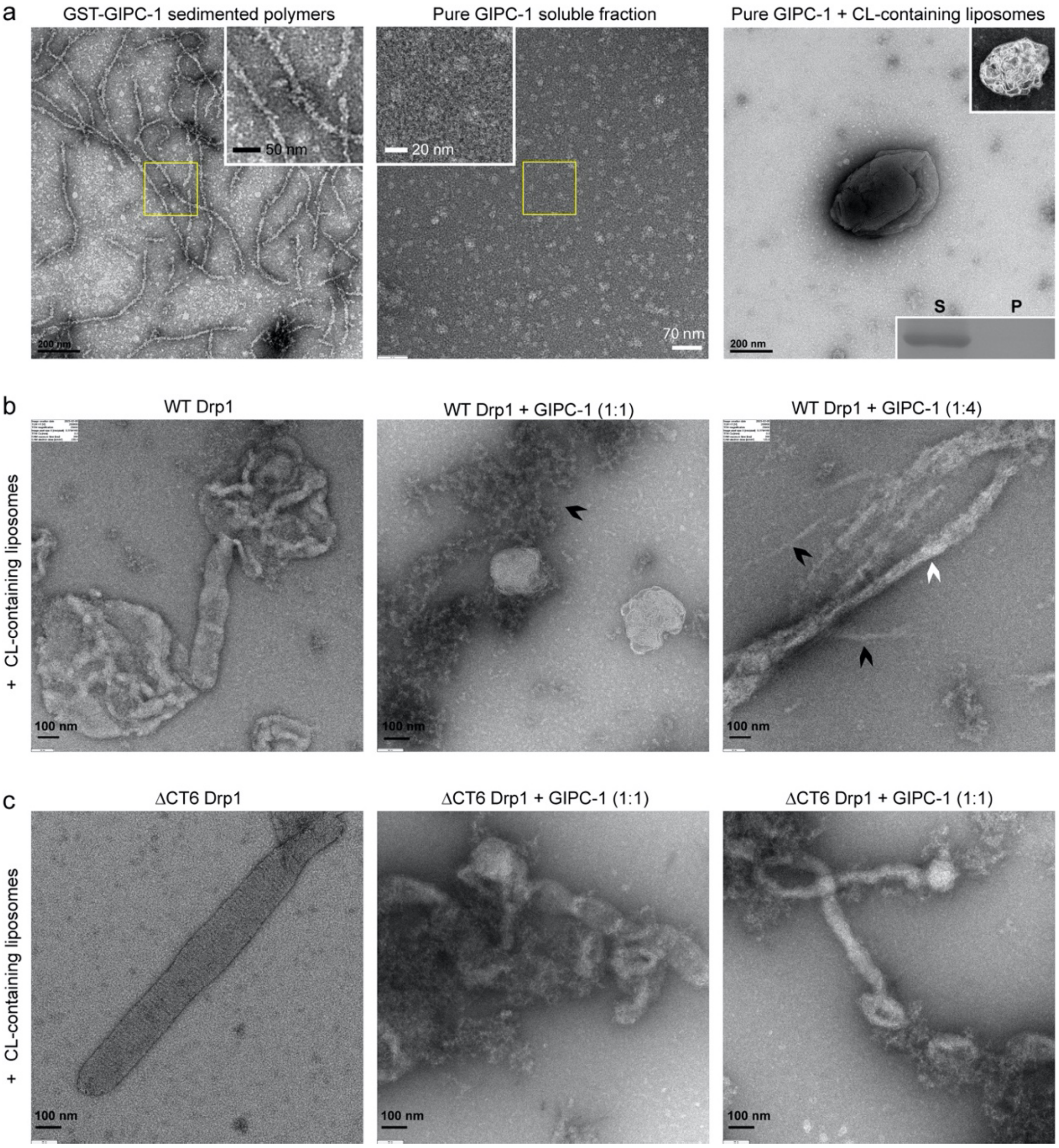

Supplementary Figure 10. GIPC-1 counteracts Drp1 self-assembly on membranes. a NS-EM of sedimented GST-GIPC-1 filamentous polymers formed at high protein concentrations during purification (left panel), purified and soluble full-length GIPC-1 at 8 μM protein concentration showing homogeneous discoid profiles averaging ~19 nm in diameter (middle panel, see inset), and purified GIPC-1 at 2 μM and 8 μM (top inset) incubated with CL-containing liposomes (50 μM lipid final) (right panel). Bottom inset shows co-sedimentation analysis of GIPC-1 in the presence of GalCer CL-NTs. Collectively, these data show that GIPC-1 is incapable of stably binding or remodeling membranes on its own. **b, c** NS-EM of WT Drp1 (**b**) and ΔCT6 Drp1 (**c**) assembled on CL-containing liposomes in the absence (left) and presence of 1:1 (middle) and 1:4 (right) molar ratio of GIPC-1. For WT Drp1, membrane tubulation was inhibited in the presence of 1:1 GIPC-1. Instead, amorphous protein assemblies (black arrowhead) in solution were observed. At the 1:4 ratio, however, linear (black arrow heads) and bundled protein filaments (white arrowhead) likely representing hybrid copolymers of Drp1 and GIPC-1 were observed. Neither Drp1 nor GIPC-1 alone formed such entities at the concentrations used. For ΔCT6 Drp1 with perturbed GIPC-

1 binding, membrane tubulation was largely preserved, although the tubes approached relatively narrow diameters in the presence of GIPC-1. Scale bar, 100 or 200 nm as indicated in the respective panels.

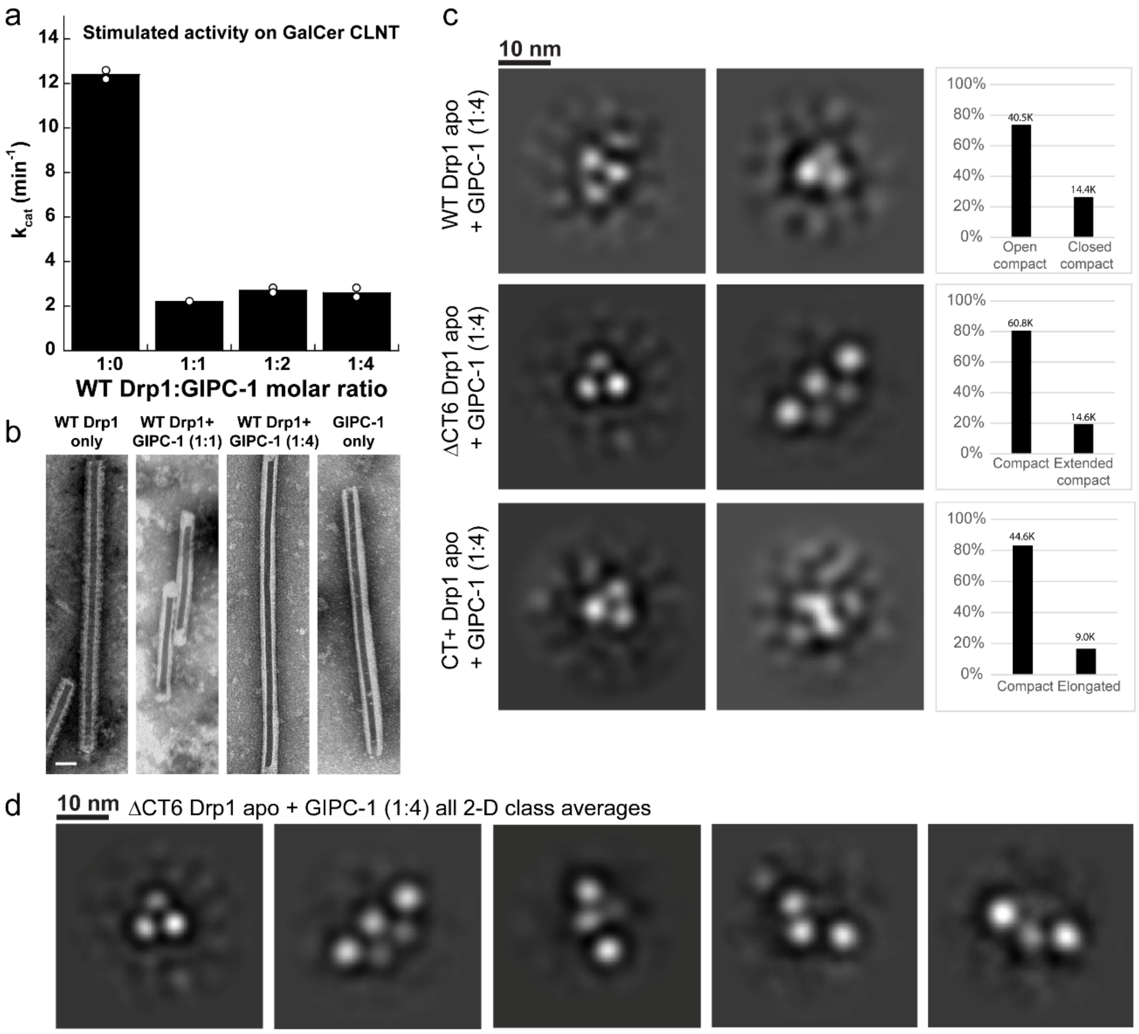

Supplementary Figure 11. GIPC-1 alters Drp1 conformational dynamics and inhibits CL-stimulated GTPase activity. **a** CL-stimulated GTPase activity of WT Drp1 on GalCer CL-NTs with increasing concentrations of GIPC-1. Individual k_{cat} values (data points) and averages (bars) from two independent experiments (n = 2) are plotted versus Drp1:GIPC-1 molar ratio. **b** NS-EM of WT Drp1 on GalCer CL-NTs upon preincubation with GIPC-1 at 1:1 and 1:4 Drp1:GIPC-1 molar ratios. GIPC-1 alone preincubated with GalCer CL-NTs is shown as control. **c, d** NS-EM 2D class averages of WT and CT variants in the apo state in the presence of a 1:4 molar ratio of GIPC-1. In contrast to WT Drp1, various other conformations besides the compact dimer, including extended chains and paired dimers were observed for the CT variants indicating the presence of additional, likely non-specific binding sites for GIPC-1. Particle numbers analyzed and % in each distinct class are shown.

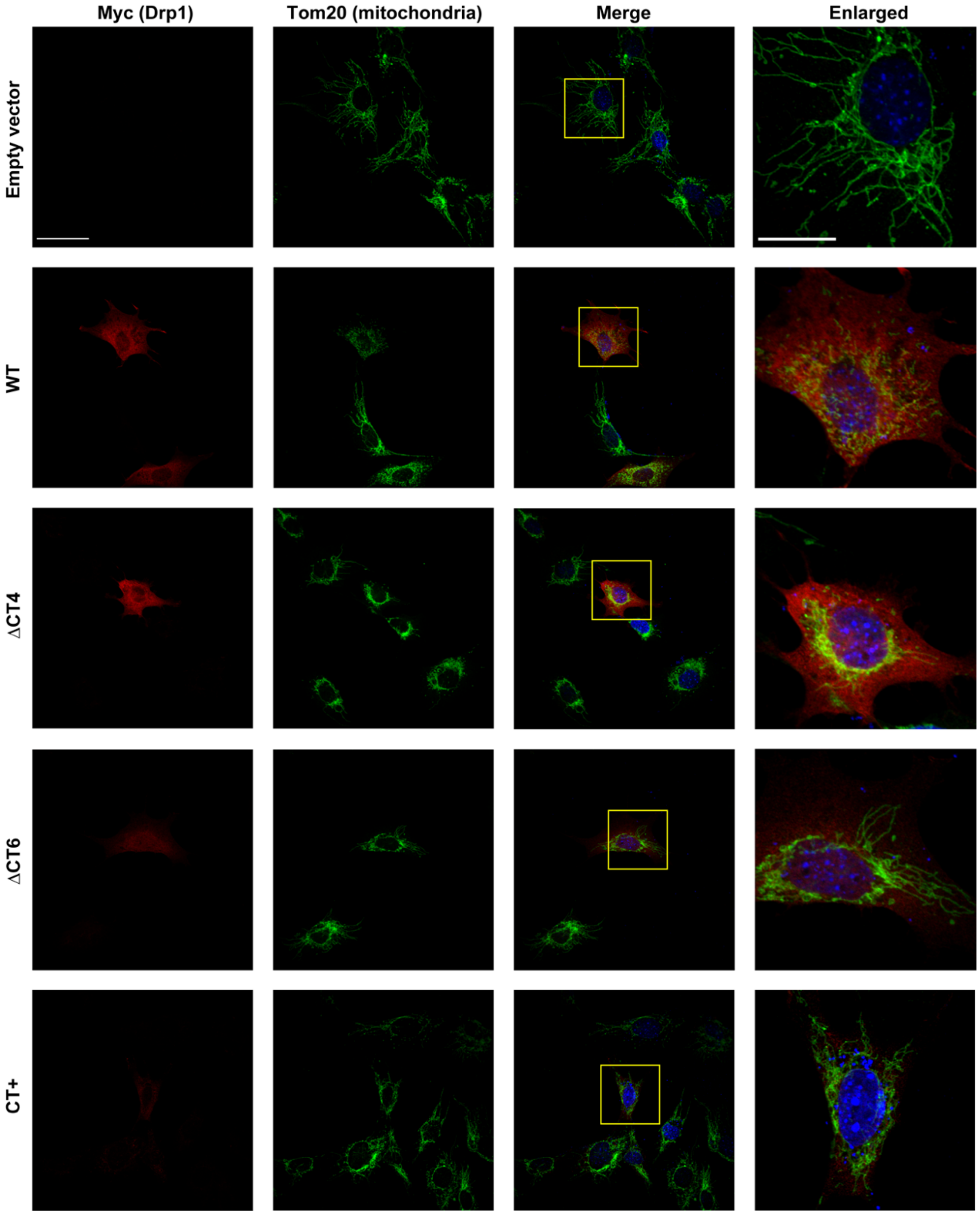

Supplementary Figure 12. CT-SLiM modifications impair mitochondrial fission in vivo. Drp1 KO MEFs overexpressing Myc-tagged WT Drp1 or CT variants were immunostained using anti-Myc and anti-Tom20 (mitochondria) antibodies. Representative images from each group are shown. Two replicate experiments were performed. Uncropped panels for Drp1 (red), mitochondria (green), and merge are displayed along with an enlarged panel of the boxed region. Scale bar for the raw and uncropped full-size images, 50 µm. Scale bar for the enlarged images, 20 µm. Zoomed images from these panels are shown in Fig. 6a.

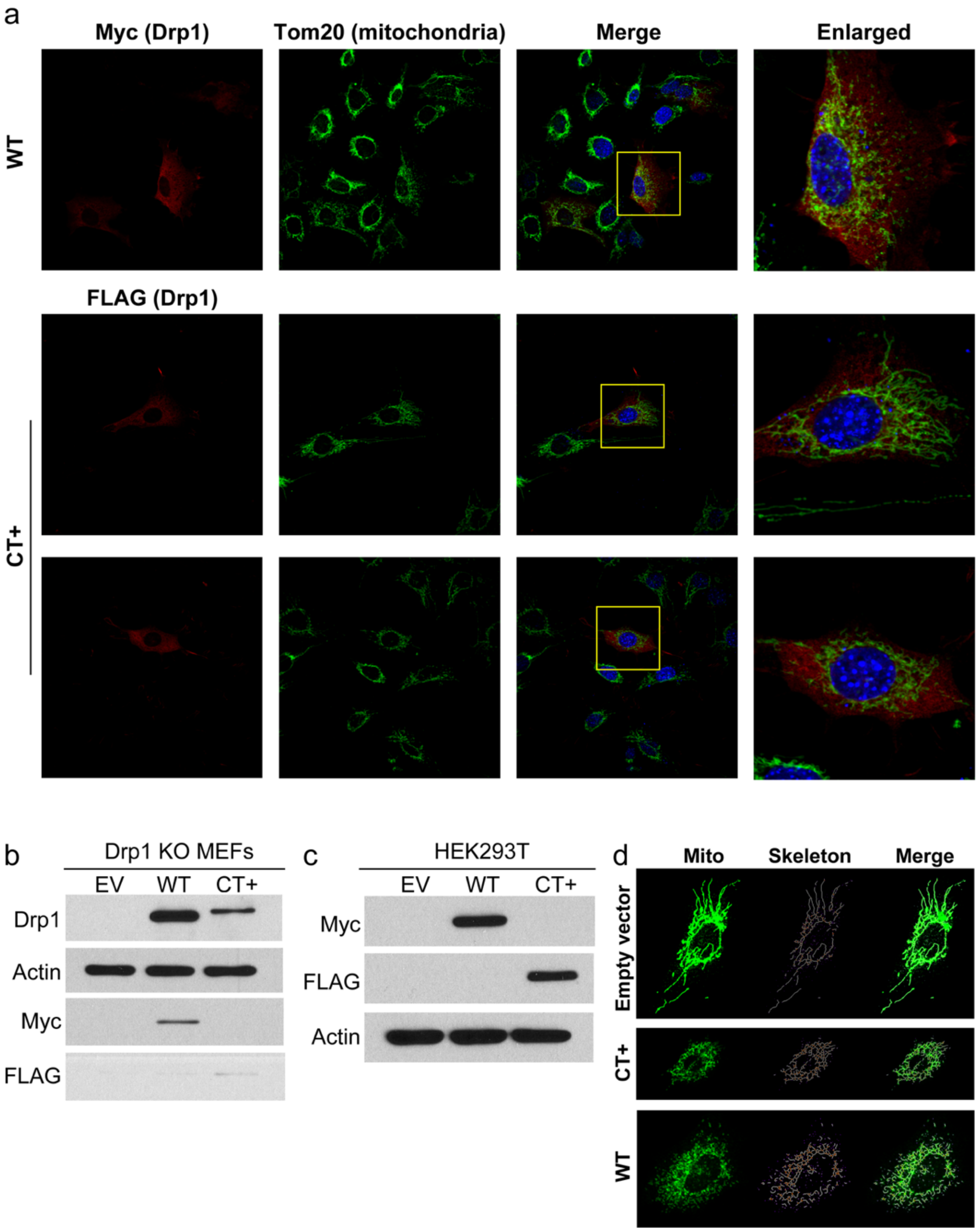

Supplementary Figure 13. CT+ Drp1 is defective in mitochondrial fission *in vivo*. **a** Same as in Fig. S12, but with CT+ Drp1 probed instead by an anti-FLAG antibody. CT+ Drp1 contains tandem Myc and FLAG tags as part of its non-native CT extension. See Methods for details. **b, c** Total lysates were harvested from Drp1 KO MEFs (**b**) and HEK293T cells (**c**) overexpressing either empty vector, Myc-tagged WT Drp1, or combined Myc+FLAG-tagged CT+ Drp1, and analyzed by western blotting using the indicated antibodies. In the case of CT+ Drp1, when expressed in both Drp1 KO MEFs and HEK293T cells, the downstream FLAG tag was more accessible to antibody staining than the upstream Myc tag positioned close to the CT-SLiM. Representative blots are shown. Actin served as loading control. One experiment for each condition. **d** Representative images of mitochondrial morphology and corresponding skeletons used for network connectivity analysis as shown in Fig. 6c.

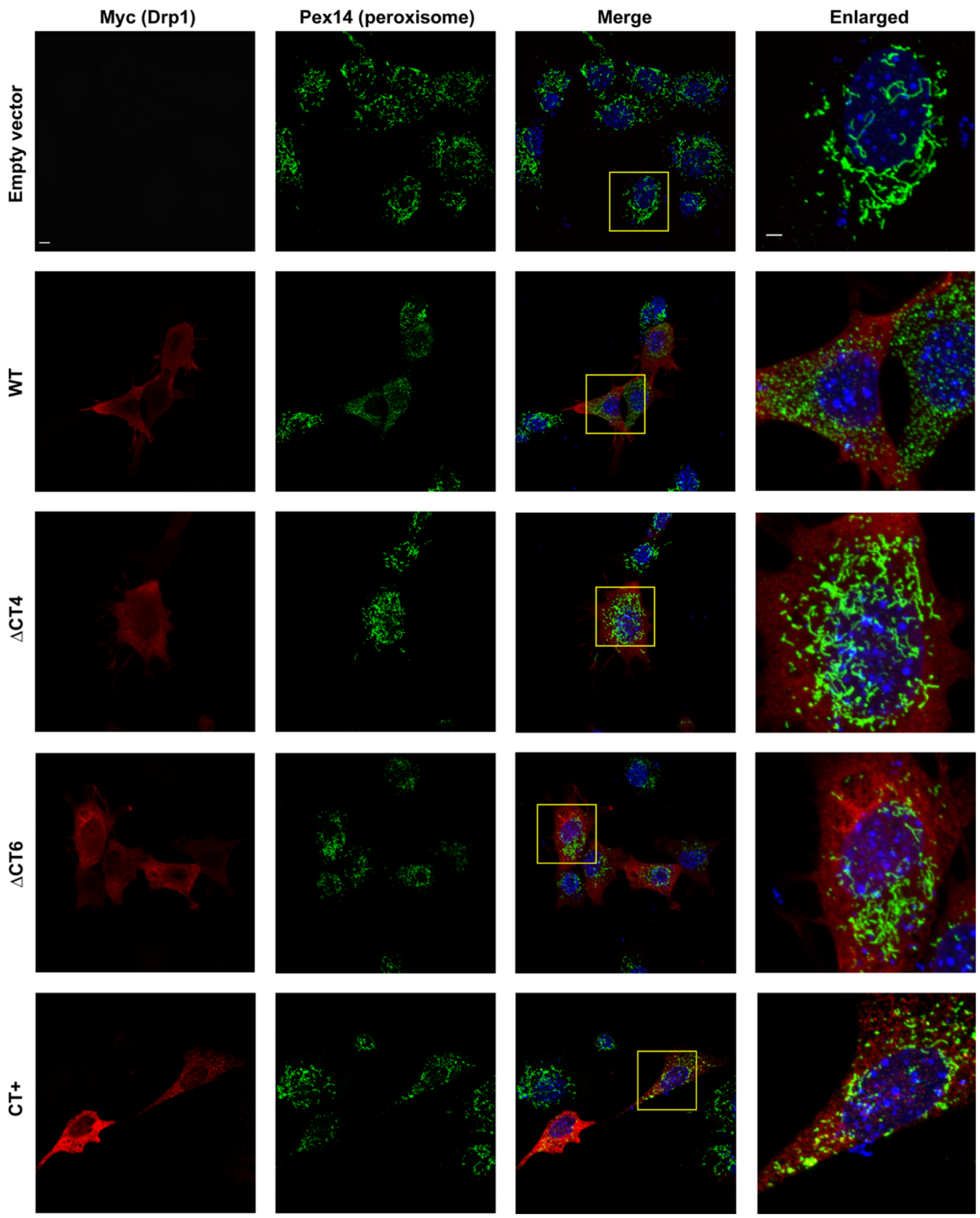

Supplementary Figure 14. CT-SLiM modifications impair peroxisomal fission *in vivo*. Drp1 KO MEFs overexpressing Myc-tagged WT Drp1 or CT variants were immunostained using anti-Myc and anti-PEX14 (peroxisomes) antibodies. Representative images from each group are shown. Uncropped panels for Drp1 (red), peroxisomes (green), and merge are displayed along with an enlarged panel of the boxed region. Scale bar for raw and uncropped full-size images, 50 µm. Scale bar for the enlarged images, 20 µm. Zoomed images from these panels are shown in Fig. 6a.

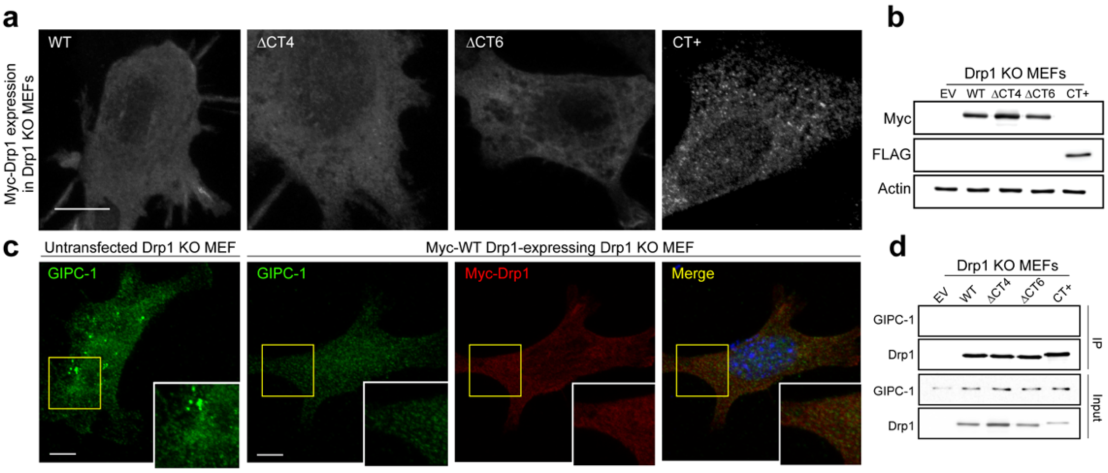

Supplementary Figure 15. CT+ Drp1 is aggregation-prone, while Drp1-GIPC-1 interactions are dynamic. a Grayscale images of overexpressed Myc-tagged WT Drp1 and CT variants in Drp1 KO MEFs showing a pronounced aggregation of CT+ Drp1, in contrast to the more diffuse and homogeneous distribution of WT Drp1 and ΔCT4/6 variants. These cells were selected from the image panels shown in Supplementary Fig. 14. Scale bar, 50 µm. **b** Total lysates were harvested from Drp1 KO MEFs overexpressing Myc-tagged WT Drp1 or CT variants and subjected to western blot analyses using the indicated antibodies. For CT+ Drp1, antibodies against the C-terminal FLAG tag was used. Actin served as loading control. This is a repetition of experiments performed in Fig. S13b with the addition of the ΔCT4 and ΔCT6 constructs. **c** (left panel) Immunostaining in untransfected Drp1 KO MEFs shows a punctiform yet homogeneous distribution of endogenous GIPC-1, whereas overexpressed Myc-tagged WT Drp1 is diffuse and does not colocalize significantly with the punctiform GIPC-1 (right panels). Scale bar, 50 µm. **d** Total lysates harvested from Drp1 KO MEFs overexpressing Myc-tagged Drp1 or CT variants were subjected to immunoprecipitation using anti-Drp1 antibodies, followed by western blotting using the anti-GIPC-1 antibodies. Input and immunoprecipitant samples are indicated. One experiment per condition.
