# Supplementary material for: Allosteric control of dynamin-related protein 1 through a disordered C-terminal Short Linear Motif
Source: Nat Commun. 2024 Jan 2;15:52. doi: 10.1038/s41467-023-44413-6 (PMC10761769; doi:10.1038/s41467-023-44413-6)
Supplement: Supplementary file 3 — Description of Additional Supplementary Files [file 41467_2023_44413_MOESM3_ESM.pdf]

Description of Additional Supplementary Files

File Name: Supplementary Movie 1
Description: NT constriction upon addition of 0.5 µM WT Drp1 in the presence of 1 mM GTP, corresponding to the kymograph in Fig. 4a. RhPE fluorescence is shown. Background was subtracted using background subtraction plugin in ImageJ.

File Name: Supplementary Movie 2
Description: NT constriction and fission upon addition of 0.5 µM DCT4 Drp1 in the presence of 1 mM GTP, corresponding to the kymograph in Fig. 4a. RhPE fluorescence is shown. Background was subtracted using background subtraction plugin in ImageJ.

File Name: Supplementary Movie 3
Description: NT constriction and fission upon addition of 0.5 µM by DCT6 Drp1 in the presence of 1 mM GTP, corresponding to the kymograph in Fig. 4a. RhPE fluorescence is shown. Background was subtracted using background subtraction plugin in ImageJ.

File Name: Supplementary Movie 4
Description: NT constriction and fission upon addition of 0.5 µM CT+ Drp1 in the presence of 1 mM GTP, corresponding to the kymograph in Fig. 4a. RhPE fluorescence is shown. Background was subtracted using background subtraction plugin in ImageJ.

File Name: Supplementary Movie 5
Description: NT constriction and fission upon addition of equimolar concentrations (0.5 µM each) of WT Drp1 and GIPC-1, corresponding to the kymograph in Fig. 5e. RhPE fluorescence is shown. Background was subtracted using background subtraction plugin in ImageJ.
